# Grayscale inversion radiographic view provided improved intra- and inter-observer reliabilities in measuring spinopelvic parameters in asymptomatic adult population

**DOI:** 10.1186/s12891-016-1269-3

**Published:** 2016-10-03

**Authors:** Weixiang Sun, Jin Zhou, Xiaodong Qin, Leilei Xu, Xinxin Yuan, Yang Li, Yong Qiu, Zezhang Zhu

**Affiliations:** 1 Department of Spine Surgery, the Affiliated Drum Tower Hospital of Nanjing University Medical School, Nanjing, China; 2 Department of Radiology, the Affiliated Drum Tower Hospital of Nanjing University Medical School, Nanjing, China; 3 The Affiliated Drum Tower Hospital of Nanjing University Medical School, Zhongshan Road 321, Nanjing, 210008 China

**Keywords:** Spinopelvic parameters, Standard view, Grayscale inversion view

## Abstract

**Background:**

Recently, a grayscale inversion view was reported to improve intra- and inter-observer reliabilities in measuring coronal curvature with Cobb and pedicle methods in scoliosis patients. However, the grayscale transformation has never been applied to the measurements of spinopelvic parameters. The purpose of this study was to compare the measurement reliabilities of the spinoplevic sagittal parameters between the ‘Standard View’ and the ‘Grayscale Inversion View’ in normal adult populations.

**Methods:**

A total of 30 asymptomatic subjects aged between 30 and 40 years were included in this study. Whole-spine posteroanterior radiographs were used to measure the spinoplevic sagittal parameters including thoracic kyphosis (TK), lumbar lordosis (LL), sagittal vertical axis (SVA), pelvic incidence (PI), sacral slope (SS) and pelvic tilt (PT) in both standard view and grayscale inversion view. Two independent observers measured the parameters twice at a 2-week interval. Intra- and inter-observer reliabilities were compared between the two radiographic views. The absolute differences between the two sets of measurements on each view were calculated and compared.

**Results:**

The intra-class correlation coefficients (ICCs) of PI, PT and SVA were greater in the grayscale inversion view than in the standard view (0.972 vs 0.817, 0.937 vs 0.833 and 0.964 vs 0.901 for observer 1, respectively; 0.990 vs 0.826, 0.995 vs 0.842 and 0.969 vs 0.919 for observer 2, respectively). Overall, the improvement of ICC was greater in parameters of sagittal pelvic alignment than in those of sagittal spinal alignment. As for the mean absolute differences between two measurements, significant differences existed between the two views in terms of PI, PT and SVA (*p* = 0.014, 0.016 and 0.011 for observer 1, respectively; *p* = 0.014, 0.025 and 0.046 for observer 2, respectively).

**Conclusions:**

A grayscale inversion view provided improved intra- and inter-observer reliabilities in measuring spinoplevic alignment when compared with a standard view. This view was more useful in subjects whose pelvic anatomical structures can’t be identified clearly on the standard X-ray view.

## Background

The measurement of spinopelvic alignment in the sagittal plane is of great importance for the evaluation of spinal sagittal balance. Obtaining measurements of spinopelvic parameters with high accuracy is crucial in establishing the surgical strategy for spinal disorders. As a most commonly used spinopelvic parameter, pelvic incidence (PI) was first described by Duval-Beaupere et al. [1] to evaluate the sagittal alignment of pelvis. Additionally, pelvic morphology (PR-S1 angle) [2] and the femoro-sacral posterior angle (FSPA) [3] were presented to serve as alternative morphologic pelvic parameters of PI in patients with a dome-shaped deformity of the sacrum. Considering that the upper edge of the pubic symphysis was easy to identify on the lateral X-ray film, Wang et al. [4] took it as an alternative landmark of the hip axis and proposed two morphologic parameters: the sacrum pubic incidence (SPI) and sacrum pubic posterior angle (SPPA). For the measurement of these parameters on a lateral view of the pelvis, it is essential to locate a midpoint between the approximate centers of the two femoral heads to establish a pelvic hip axis [2]. However, due to the occlusion from soft tissue, it is often difficult to detect the profile of round femoral head and the posterior border of sacrum on radiographs, thereby limiting the ability to determine these morphologic parameters.

A grayscale inversion view in PACS (Picture Archiving and Communication System; Marosis; Infinitti) has been widely used to detect small pulmonary nodules in chest radiographs [5–10]. This grayscale transformation, by which the images in radiographs were viewed as dark structures on a bright background instead of standard bright on dark presentation, was reported to improve the observer’s ability to detect shadows of pathologic pulmonary nodules in a chest radiograph [10, 11]. Recently, a grayscale inversion view was reported to improve intra- and inter-observer reliabilities in measuring coronal curvature with Cobb and pedicle methods in scoliosis patients [12]. However, the grayscale transformation has never been applied to the measurements of spinopelvic parameters.

The purpose of this study is to compare a grayscale inversion view with a standard view in terms of inter- and intra-observer reliabilities of the spinoplevic radiologic measurements. Our hypothesis is that a grayscale inversion view would provide higher intra- and inter-observer reliabilities than a standard view.

## Methods

### Patient selection

Under the approval of local Institutional Review Board, the standing X-rays of 30 asymptomatic subjects aged between 30 and 40 years were randomly selected from the database. This database belongs to the department of spine surgery of our hospital, which was composed of asymptomatic volunteers recruited from a community. Permission has been obtained to access the database. The following inclusion criteria were used: 1. with no history of spinal pathology or deformity. 2. with no history of pelvic, hip as well as lower limbs disorders. Subjects were excluded from our study if they had any positive radiographic finding or a history of low back pain for a minimum of 3 consecutive months.

### Radiographic measurements

**Fig. 1:**
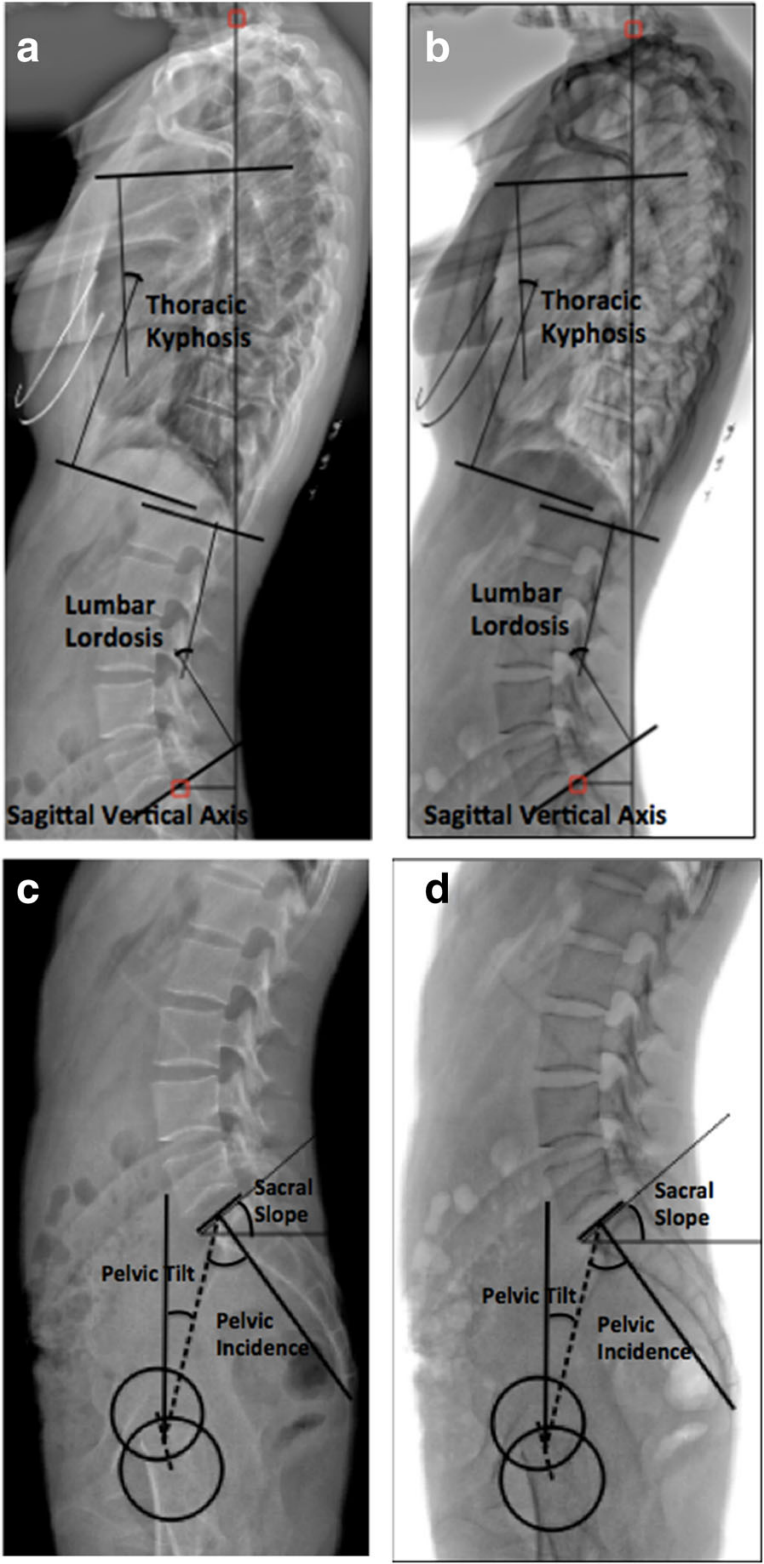
Methods of measurements of spinopelvic parameters on the standard view (**a**, **c**) and the grayscale inversion view (**b**, **d**). Thoracic kyphosis was defined as the value of angle between the upper endplate of the T5 and the lower endplate of T12. Lumbar lordosis was defined as the value of angle between the superior endplate of L1 and the superior endplate of S1. Sagittal vertical axis was defined as the horizontal distance between the postero-superior corner of the sacrum and the C7 plumb line. Pelvic incidence was defined as the value of the angle between the line perpendicular to the superior plate of S1 at its midpoint and the line connecting this point to the center of the line connecting the centers of the femoral heads. Sacral slope was defined as the value of the angle between the superior plate of S1 and a horizontal line. Pelvic tilt was defined as the value of the angle between the vertical and the line connecting the midpoint of the sacral plate to the axis of the femoral heads

**Fig. 2:**
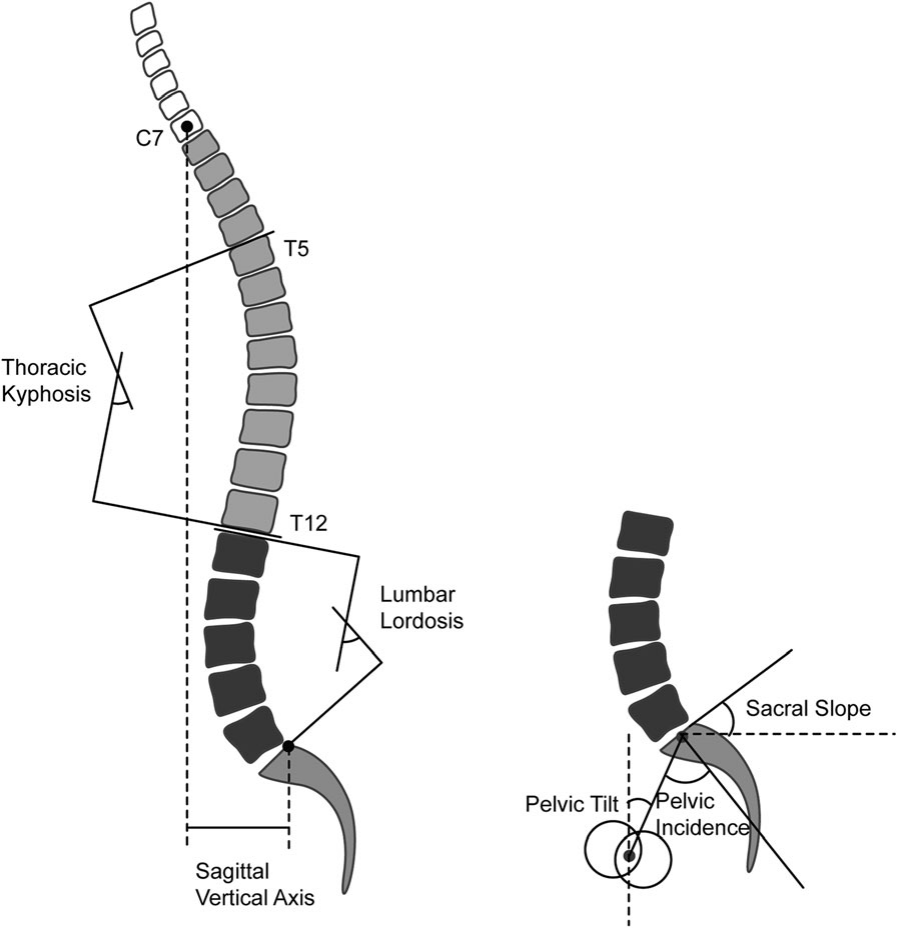
An illustration of the spine showing the lines and angles

All the radiographs of the pelvis and entire spine were obtained with each subject standing in a comfortable position. The subjects were instructed to keep the knees fully extended, with the hips perpendicular to the X-ray cassette. A total of 6 sagittal parameters were measured in the radiographs, including thoracic kyphosis (TK), lumbar lordosis (LL), sagittal vertical axis (SVA), pelvic incidence (PI), sacral slope (SS) and pelvic tilt (PT) (Figs. 1a, b, c, d and 2). The TK was defined as the value of angle between the upper endplate of the T5 and the lower endplate of T12. The LL was defined as the value of angle between the superior endplate of L1 and the superior endplate of S1. The SVA was defined as the horizontal distance between the postero-superior corner of the sacrum and the C7 plumb line. When the sacral posterior corner landed in front of the C7 plumb line, the value was defined as positive. The PI was defined as the value of the angle between the line perpendicular to the superior plate of S1 at its midpoint and the line connecting this point to the center of the line connecting the centers of the femoral heads. The SS was defined as the value of the angle between the superior plate of S1 and a horizontal line. The PT was defined as the value of the angle between the vertical and the line connecting the midpoint of the sacral plate to the axis of the femoral heads.

Two attending surgeon who had more than five years of experience in terms of spinal radiographic interpretation independently measured all variables twice at a 2-week interval on two views (standard and inversion view) of 30 X-rays images. Surgimap software (version 2.1.2) was used to view radiographic images and measure the parameters. Therefore, there were a total of 1440 (6 × 2 × 2 × 2 × 30) measurement trials, all of which can be considered blinded because a 2-week interval was long enough for observer to clear the memory of the parameters measured at first.

### Evaluation of measurements reliabilities

After obtaining all the parameters described above, the intra- and inter-observer reliabilities were calculated. Two-times measurements with 2-week interval for each observer enabled us to assess the intra-observer reliability. The intra-class correlation coefficients (ICCs) with its 95 % confidence interval were calculated for each parameter. The ICCs were compared between the standard view and the grayscale inversion view for both observers. Besides, the inter-observer reliabilities were also assessed, with the ICCs calculated for each parameter. Furthermore, the absolute differences between the two sets of measurements on each view were calculated and compared using Paired-Samples T Test. The statistical software SPSS (Version 19.0) was used for all statistical analyses. *P* < 0.05 was considered statistically significant.

## Results

As shown in Tables 1 and 2, the ICCs which represent the intra-observer reliabilities were greater in a grayscale inversion view than in a standard view in all parameters for observer 1. The ICC was increased from 0.901 in a standard view to 0.964 in a grayscale inversion view in SVA, from 0.817 to 0.972 in PI, and from 0.833 to 0.937 in PT. Overall, the improvement of ICC was greater in parameters of sagittal pelvic alignment than in those of sagittal spinal alignment. Similar trends were observed for the other observer (Table 2).

**Table 1:** Intra-observer reliabilities of the observer 1

	Standard view		Grayscale inversion view	
	ICC^a^	95 % CI^b^	ICC^a^	95 % CI^b^
Thoracic kyphosis	0.921	0.843–0.967	0.947	0.869–0.967
Lumbar lordosis	0.954	0.911–0.978	0.959	0.922–0.983
Sagittal vertical axis	0.901	0.853–0.924	0.964	0.937–0.981
Pelvic incidence	0.817	0.624–0.907	0.972	0.941–0.991
Pelvic tilt	0.833	0.647–0.916	0.937	0.889–0.973
Sacral slope	0.941	0.853–0.969	0.956	0.897 - 0.971

ICC^a^ indicates inter- and/or intra-class correlation coefficient; CI^b^ indicates confidence interval

**Table 2:** Intra-observer reliabilities of the observer 2

	Standard view		Grayscale inversion view	
	ICC^a^	95 % CI^b^	ICC^a^	95 % CI^b^
Thoracic kyphosis	0.916	0.823–0.960	0.941	0.875–0.972
Lumbar lordosis	0.960	0.917–0.981	0.968	0.932–0.985
Sagittal vertical axis	0.919	0.868–0.934	0.969	0.935–0.985
Pelvic incidence	0.826	0.635–0.917	0.990	0.979–0.995
Pelvic tilt	0.842	0.668–0.925	0.995	0.989–0.998
Sacral slope	0.933	0.859–0.968	0.994	0.987–0.997

ICC^a^ indicates inter- and/or intra-class correlation coefficient; CI^b^ indicates confidence interval

Table 3 summarized the ICCs indicating the inter-observer reliabilities of the two observers. At the first measurement, the ICC was increased from 0.863 in a standard view to 0.983 in a grayscale inversion view in PI, from 0.815 to 0.917 in PT, and from 0.918 to 0.983 in SVA. On the whole, the improvement was more obvious in parameters of sagittal pelvic alignment than in parameters of sagittal spinal alignment. Similar trends were also observed for the second measurement.

**Table 3:** Inter-observer reliabilities of the observer 1 and observer 2

	Parameters	Standard view		Grayscale inversion view	
		ICC^a^	95 % CI^b^	ICC^a^	95 % CI^b^
1^st^ time	Thoracic kyphosis	0.892	0.774–0.949	0.897	0.784–0.951
	Lumbar lordosis	0.968	0.933–0.985	0.956	0.907–0.979
	Sagittal vertical axis	0.918	0.875–0.934	0.983	0.973–0.991
	Pelvic incidence	0.863	0.777–0.950	0.983	0.965–0.992
	Pelvic tilt	0.815	0.612–0.912	0.917	0.826–0.960
	Sacral slope	0.888	0.764–0.947	0.933	0.858–0.968
2^nd^ time	Thoracic kyphosis	0.945	0.884–0.974	0.932	0.857–0.968
	Lumbar lordosis	0.948	0.891–0.975	0.937	0.867–0.970
	Sagittal vertical axis	0.913	0.863–0.937	0.993	0.985–0.997
	Pelvic incidence	0.874	0.734–0.940	0.941	0.913–0.957
	Pelvic tilt	0.895	0.779–0.950	0.926	0.845–0.965
	Sacral slope	0.925	0.843–0.965	0.984	0.967–0.992

ICC^a^ indicates inter- and/or intra-class correlation coefficient; CI^b^ indicates confidence interval

Table 4 showed the mean absolute differences between two measurements of the sagittal parameters. For the parameters of sagittal pelvic alignment, significant differences existed between the two views in terms of PI and PT (*P* = 0.014 and 0.016 for observer 1, respectively; *p* = 0.014 and 0.025 for observer 2, respectively). However, for the parameters of sagittal spinal alignment, there were no significant differences between the two modes of views except for SVA (*P* = 0.011 for observer 1 and 0.046 for observer 2). Similar results were observed for the other observer.

**Table 4:** The absolute difference between two measurements

	Parameters	Standard view	Grayscale inversion view	*P* value
		Mean ± S.D.	Mean ± S.D.	
Observer 1	Thoracic kyphosis	2.89 ± 2.07	2.17 ± 2.36	0.214
	Lumbar lordosis	2.84 ± 1.91	2.21 ± 1.63	0.175
	Sagittal vertical axis	3.78 ± 3.01	2.07 ± 1.83	0.011
	Pelvic incidence	3.23 ± 2.19	1.79 ± 2.21	0.014
	Pelvic tilt	2. 89 ± 3.14	1.23 ± 1.91	0.016
	Sacral slope	0.34 ± 2.83	0.56 ± 1.47	0.707
Observer 2	Thoracic kyphosis	2.87 ± 2.96	1.94 ± 2.07	0.164
	Lumbar lordosis	3.11 ± 2.24	2.52 ± 2.24	0.160
	Sagittal vertical axis	2.47 ± 2.38	1.48 ± 1.19	0.046
	Pelvic incidence	3.16 ± 2.27	1.84 ± 1.73	0.014
	Pelvic tilt	2.63 ± 2.31	1.43 ± 1.69	0.025
	Sacral slope	0.53 ± 1.45	0.41 ± 1.39	0.744

Values are expressed as mean ± standard deviation for continuous variables

## Discussions

Sagittal pelvic morphology plays an important role in maintaining a normal biomechanical stress at the lumbo-sacral junction [13] and an adequate posture on sagittal plane of humans. It can also help understand the biomechanical pathogenesis of several spinal and pelvic diseases, such as lumbar spondylolisthesis [13, 14] and low back pain [15, 16]. To date, different parameters have been proposed to describe the pelvic morphology, among which PI is an outstanding anatomic parameter illustrating pelvic orientation [17] and guiding surgical decision [13].

To assess the value of PI accurately, it is crucial to identify the femoral heads and the superior endplate of S1 on lateral X-ray films. However, it is sometimes difficult to identify the femoral heads axis and measure the PI on the standard view X-ray image, especially in patients with severe hip osteoarthritis or ankylosing arthritis, whose femoral heads are not round in shape or in fusion with acetabulum [18, 19]. Thus, it is of great interest to improve the accuracy of measurements of spinopelvic parameters in order to provide a solution for accurate evaluation of spinopelvic alignment.

A grayscale inversion view of X-ray films was first developed to detect the nodule on chest radiography [5, 6, 9, 10]. It was reported that significantly higher specificity in pulmonary nodule detection could be obtained by the grayscale inversion view as compared with a standard display [10, 11]. Recently, the grayscale inversion view was used to improve the measurement of coronal curvatures in patients with scoliosis [12]. The grayscale inversion radiographic view was confirmed to provide improved intra- and inter-observer reliabilities in radiographic measurements. It is known from eye detection theory that the optical contrast perception would increase when a dark structure is presented on a bright background [9, 11, 20, 21]. When a target predominantly surrounded by an area of higher luminance (grayscale inversion display), the target should be more easily detected than those predominantly surrounded by an area of lower luminance (traditional display) [11, 17, 22].

**Fig. 3:**
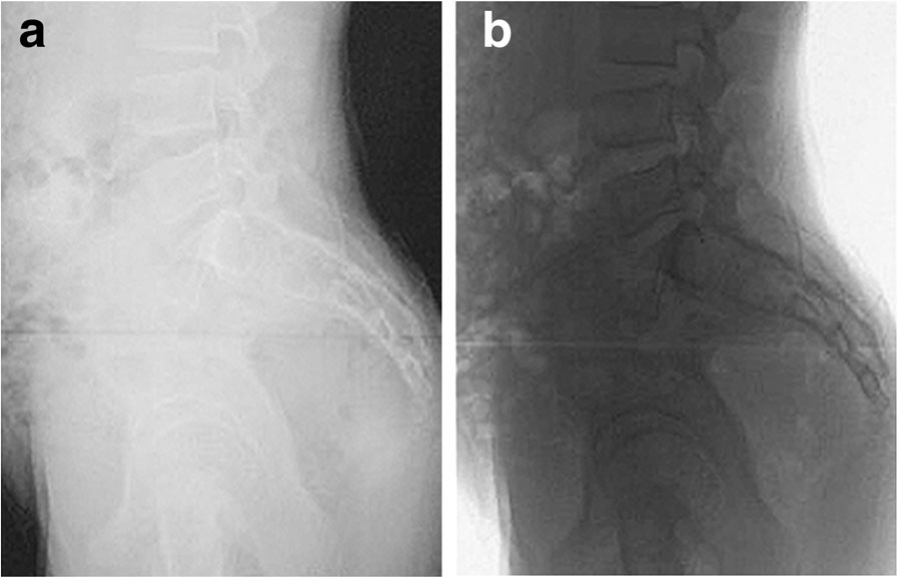
An illustrative figure demonstrating the profiles of two femoral heads and the postero-superior corner of the sacrum on the standard view (**a**) and the grayscale inversion view (**b**). Compared with the standard view, the profiles of femoral heads and the postero-superior corner of the sacrum look much distinct on the grayscale inversion view

In our study, we aimed to compare the measurement reliabilities of the sagittal aligment between the two radiographic views in normal populations. We found that the intra- and inter-observer reliabilities were higher in the grayscale inversion view than in the standard view for all parameters, especially in sagittal pelvic parameters such as PI and PT. Consistently, the mean absolute differences between two measurements were significant less on the grayscale revision view than on the standard view in measurements of sagittal pelvic parameters. By contrast, no significant difference can be found when comparing the two views in measuring sagittal spinal parameters. It appears that grayscale inversion view can specifically improve the measurement of sagittal pelvic parameters. The center of femoral heads and the postero-superior corner of the sacrum used as landmarks in PI and PT look much distinct by grayscale inversion (Fig. 3a, b), which make it easy to draw a line along these landmarks. Due to the shared landmark point with PI—the postero-superior corner of the S1, the measurement reliabilities of SVA was also improved when the grayscale inversion view was used.

Several limitations still exist in the current study. According to the definition, ICC of more than 0.8 indicates good reliability. In our study, all ICCs are more than 0.8, while there is no statistical test applicable for the comparison of the two ICCs of the standard view and the grayscale inversion view. Therefore, it is somewhat farfetched to conclude that improved intra- and inter-observer reliabilities can be obtained from the grayscale inversion view, even though they are numerically different. However, the grayscale inversion view produced consistently greater ICCs than the standard view no matter which parameter was measured. Based on our results, consistently less variation on the grayscale view can be confirmed as compared with the standard view.

## Conclusions

A grayscale inversion radiographic view provided improved intra- and inter-observer reliabilities in measuring spinoplevic alignment when compared to a standard view in normal populations. It could be a good option for measurement of sagittal pelvic parameters in subjects whose pelvic anatomical structures can’t be identified clearly on the standard X-ray view.

## Abbreviations

ICCs: Intra-class correlation coefficients
LL: Lumbar lordosis
PI: Pelvic incidence
PSPA: Femoro-sacral posterior angle
PT: Pelvic tilt
SPI: Sacrum pubic incidence
SPPA: Sacrum pubic posterior angle
SS: Sacral slope
SVA: Sagittal vertical axis
TK: Thoracic kyphosis
